# Vestibular Aging Process from 3D Physiological Imaging of the Membranous Labyrinth

**DOI:** 10.1038/s41598-020-66520-w

**Published:** 2020-06-15

**Authors:** Hisaya Tanioka, Sayaka Tanioka, Kimitaka Kaga

**Affiliations:** 1 Tanioka Clinic, Department of Radiology, Tokyo, 113-0021 Japan; 2 National Institute of Sensory Organs, National Tokyo Medical Center, Tokyo, 152-8902 Japan

**Keywords:** Computational neuroscience, Motor control, Sensory systems, Translational research

## Abstract

There is no three-dimensional (3D) technique to study the microanatomical structures of the *in vivo* 3D vestibular membranous labyrinth. Recent two MRI methods using a contrast agent can only depict the low-resolution imaging of endolymphatic hydrops. Therefore, we provide the new precise volume rendering algorithms to create the *in vivo* 3D vestibular membranous labyrinth images from high-resolution temporal bone low-dose CT data. We also ascertain whether the created 3D microstructure images are reliable in anatomical findings. Secondary, we will analyze the age-related changes of the vestibular membranous labyrinth. These created 3D membranous vestibular images were almost consistent with the appearance, dimensions, areas, and angles from those acquired in previous histological works. The age-related image changes showed the enlarged saccule in females, the enlarged utricle in males, and the dilated tendency of the lateral semicircular duct. These results may correlate to the findings of the previous physiological works on cervical and ocular vestibular evoked myogenic potentials, and gait studies. The age-related balance disorders may be associated with the enlargement of each membranous organ in the vestibule. This new imaging technique now enables visualizing microanatomical changes in the *in vivo* membranous vestibulum, and these created 3D images may suggest physiological information.

## Introduction

Although amazing progress and spread of CT and MRI have resulted in steadily increasing in volume and improvement in the accuracy of the anatomical image information of the temporal bone region^1–5^, our knowledge of the *in vivo* three-dimensional (3D) membranous vestibular apparatus under normal and pathological conditions is unclear. Two recent methods have MR imaging enabled the depiction of endolymphatic hydrops^3–5^. One is to inject contrast agent intratympanically through the tympanic membrane for *in vivo* confirmation of endolymphatic hydrops^3^. The other is a delayed 3D real inversion recovery scan 4 hours after intravenous contrast agent administration^4^. The purpose of these methods is to image the peri- or endolymphatic space. These created MR images using a head coil have too large a field of view (FOV) to delineate the inner ear minute structures^3–5^. Therefore, it seems impossible to delineate the microstructure of the membranous labyrinth from these images due to low-resolution. The low-resolution images cannot be reconstructed the tolerated 3D microanatomical images. And these methods have to use gadolinium-based contrast agents (GBCAs). The potential for and mechanisms of toxicity is gradually becoming clear^6–8^. Thus, we have no simple method to study the *in vivo* 3D vestibular membranous labyrinth of the microanatomical information. Meanwhile, the rapid technology of computer medical graphics has also along with the development of CT and MRI. A volume rendering technique has been developed since 1980. Especially in CT, X-ray imaging has been developed to study anatomical digital imaging consisted of X-ray absorption values. Digital medical image data are manipulated in a matrix of volume elements called voxels. An image is constructed by analyzing each voxel and projecting the result on a two-dimensional (2D) surface subdivided into picture elements called pixels. CT values derived from the X-ray absorption value can be assigned to the objects. Volume rendering images are generated from discrete objects of the volume data obtained from X-ray CT. Volume rendering is supposed to use all voxel values, but in reality, it is not so. Given the tissue properties and information about the perspective of the viewer, the resulting image shows the result of how light behaves as it passes through the volume voxels. With a model defined in terms of opacity, rays of light will penetrate the tissue in varying degrees. At each point, a certain amount of light is reflected and the rest is transmitted to the next layer where the same process occurs. In the end, the viewer sees an image which is the sum of reflections from each layer. Therefore, volume rendering can be roughly divided into the following four^9–11^. (1) Showing the highest value [MIP: maximum intensity projection], or the lowest value [MinIP: minimum intensity projection]. (2) Summing the voxel values from the backside [Ray Sum: summation projection]. (3) The voxel values change pseudocolor and transparency. (4) Prioritizing the voxel values in the abrupt changed voxel values areas such as a surface-based method [like Surface rendering]. These algorithms can be freely combined. And in this study, we combine the algorithms under the threshold method. The threshold method extracts a region of interest by selecting a range of voxel values that presents a specific tissue or anatomical feature. In volume rendering, the most important property is opacity. Opacity measures the degree to which a voxel obstructs visualization of the light emitted by the voxels behind it. Opacity allows you to hide certain objects or make them transparent. A voxel opacity curve algorithm can be adjusted to render tissues within a certain density range^11^. Volume rendering allows the simultaneous display of an object with different properties. Thereby, a volume rendering technique is an important role in 3D medical imaging as it includes most of the original 3D data in the visualization process rather than the converted 2D digital CT image data from volume data. The precise volume rendering technique is suitable to delineate the complex anatomy of the vestibular membranous labyrinth. So, we make the new algorithm to create the *in vivo* 3D membranous labyrinth by combining several volume rendering algorithms. And, we also assume that our created microanatomical 3D images may represent the imaging changes of the vestibular membranous labyrinth associated with the physiological phenomena at that site. Therefore, we think that it is possible to clarify whether or not this hypothesis holds by comparatively examining the relationship between the age-related image changes and the physiological aging phenomenon. Because we maybe find that there are physiological differences in the age-related changes from several papers^12–18^.

This study aims to develop and use the new volume rendering algorithms that allow for the visualization of *in vivo* 3D vestibular membranous labyrinth from clinical high-resolution temporal low-dose CT data, to analyze the accuracy of the created 3D images in comparison with the previous microanatomy books and papers^19–28^, to investigate whether the membranous vestibular CT values and dimensions change with age and whether the created 3D microanatomical image may be related to the aging process of the membranous labyrinth and physiological phenomena.

## Results

Radiation doses from CT scans were recorded as displayed on the CT monitor (from the manufacturer’s data; General Electric Medical Systems). The range of dose length product (DLP) was 40 mGy/cm to 55 mGy/cm. The voxel volume of the 3D created image was 0.18 × 0.18 × 0.5 mm^3^.

### Imaging parameter, modeling algorithm, and qualitative image evaluation

The relationship between the membranous vestibular CT value and the opacity value was a significant correlation (Y = 0.181X + 20.430: Y = the percentage opacity value, X = the membranous vestibular CT value, r: 0.771, r^2^: 0.595, p < 0.001). We defined that a range of the threshold value was −100 to 160 HU, the opacity value was 40% of the average, and the brightness value was 100%.

The different transparent 3D membranous microanatomical images were created from four different volume rendering algorithms. The relationship between the algorithm and microanatomical image was as follows.

**Figure 1:**
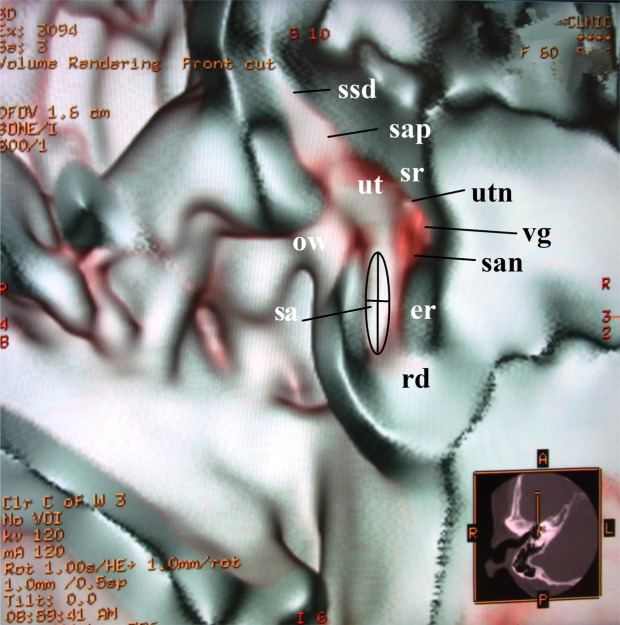
Anterior cut view of the utricle and the saccule in a 60-year-old female. This image is created from an upward curve with a merged color algorithm under 40% opacity value. The anterolateral aspect of the utricle is seen in the elliptical recess. The saccule is located in the spherical recess in the anteromedial part of the vestibule at the level of the oval window. The anterior ampulla connected with the utricle looks like a bud shape. The running nerves can be seen in this image. The dimensions of the utricle are measured as in this figure. Abbreviations: ut = utricle, sa = saccule, sap = superior ampulla, ssd = superior semicircular duct, rd = ductus reuniens, ow = oval window, er = elliptical recess, sr = spherical recess, utn = utricular nerve, vg = vestibular ganglion, san = saccular nerve.
  This algorithm created the views of the elliptical and spherical recess, soft tissues, and nerves on the saccule and the utricle.
B. An upward opacity curve combining a step and a merged color algorithm (Fig. 2)

A. An upward opacity curve using a merged color algorithm (Fig. 1)

**Figure 2:**
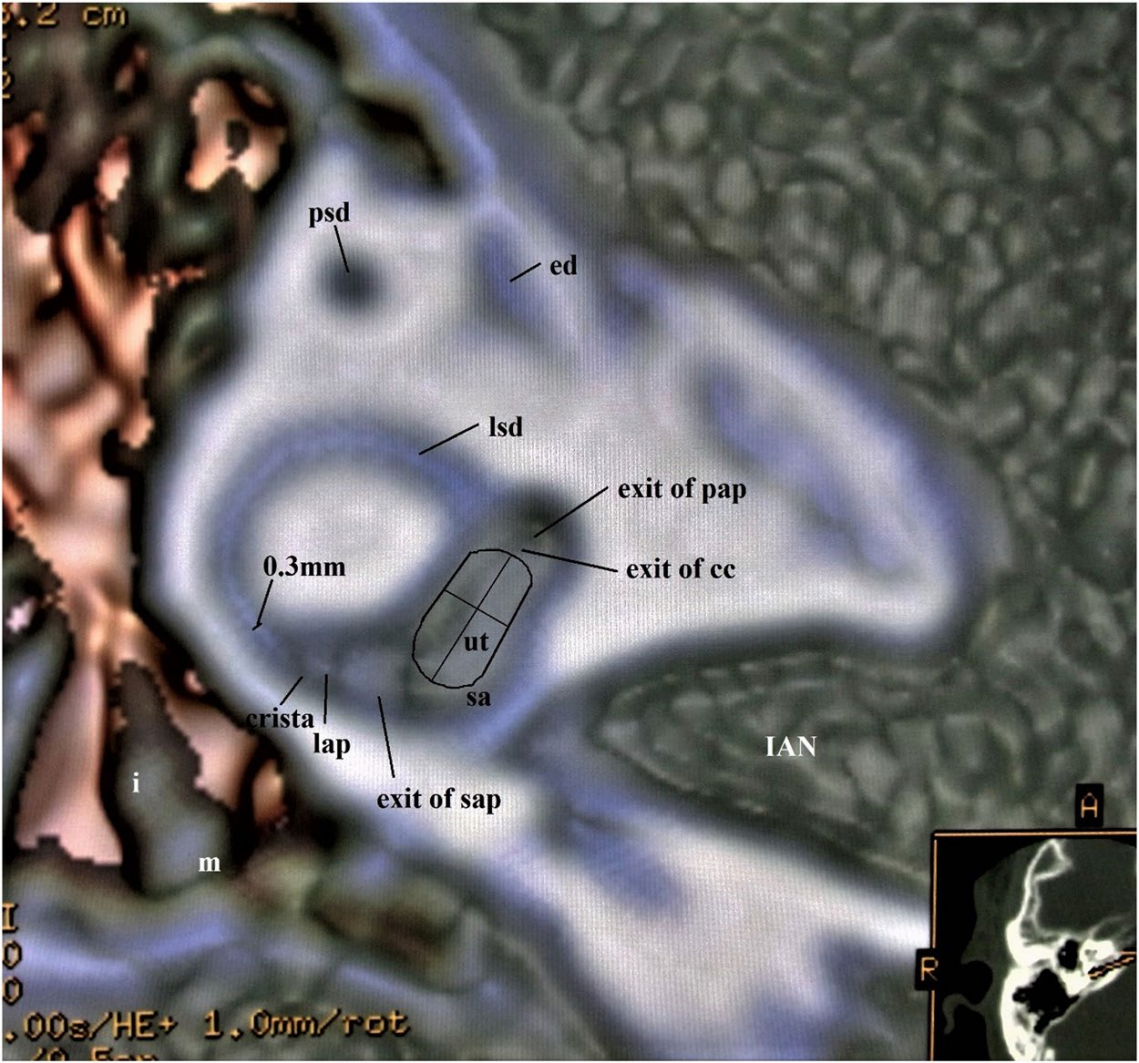
Superior view of the membranous vestibule in a 45-year-old male. This image is created from an upward curve with a step and a merged algorithm under 40% opacity value. The portion of the utricle lies approximately in the plane of the lateral semicircular duct. The crista ampullaris within the lateral ampulla shows a small triangle. The membranous structures of the vestibule are well shown in the mutual relationship. The width of the lateral semicircular duct was 0.3 mm. The line extending from the anterior to the posterior tip represents the width, and the cross line represents the length of the utricle. The area is measured along the utricular contour. Abbreviations: ut = utricle, sa = saccule, sap = superior ampulla, pap = posterior ampulla, lap = lateral ampulla, lsd = lateral semicircular duct, psd = posterior semicircular duct, ed = endolymphatic duct, IAN = Internal Auditory Nerve, m = malleus, i = incus.
  The surface contours and shapes of the utricle, saccule, lateral semicircular duct, ampullae, and ossicles were reconstructed.
C. A trapezoid opacity curve using a non-merged colored algorithm (Fig. 3)

**Figure 3:**
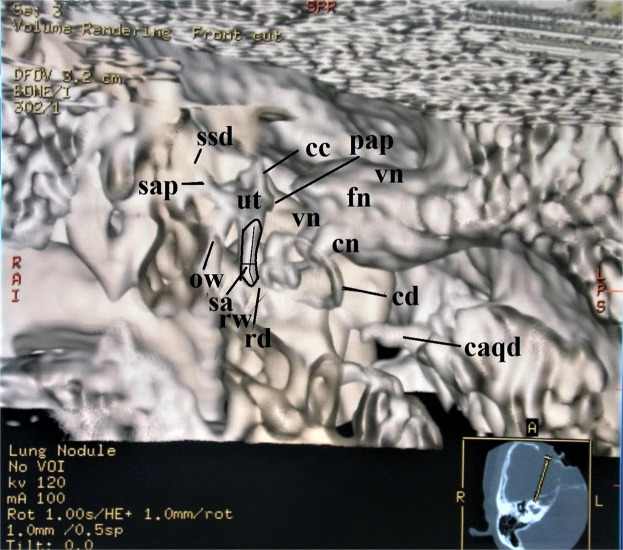
Anterosuperior view of the membranous labyrinth in a 62-year-old female. A trapezoid curve with a non-merged color algorithm under 40% opacity value makes this image. The length of the saccule is along the line and the width is the cross line. The area of the saccule is along the saccular contour. The saccule, the utricle, and the cochlear duct lie in the 3D plane. Abbreviations: sa = saccule, ut = utricle, rd = ductus reuniens, cd = cochlear duct, cc = common crus, sap = superior ampulla, ssd = superior semicircular duct, pap = posterior ampulla, ow = oval window, rw = round window, cn = cochlear nerve, vn = vestibular nerve, fn = facial nerve, caqd = cochlear aqueduct.
  This algorithm created the surface contours and shapes of the saccule, utricle, ampullae, and cochlear duct from the anterosuperior view of the membranous labyrinth. The membranous vestibular aspect showed the utricle looked like an acorn or a shield, and the saccule resembled a cucumber.
D. A downward opacity curve using a merged colored algorithm (Fig. 4)

**Figure 4:**
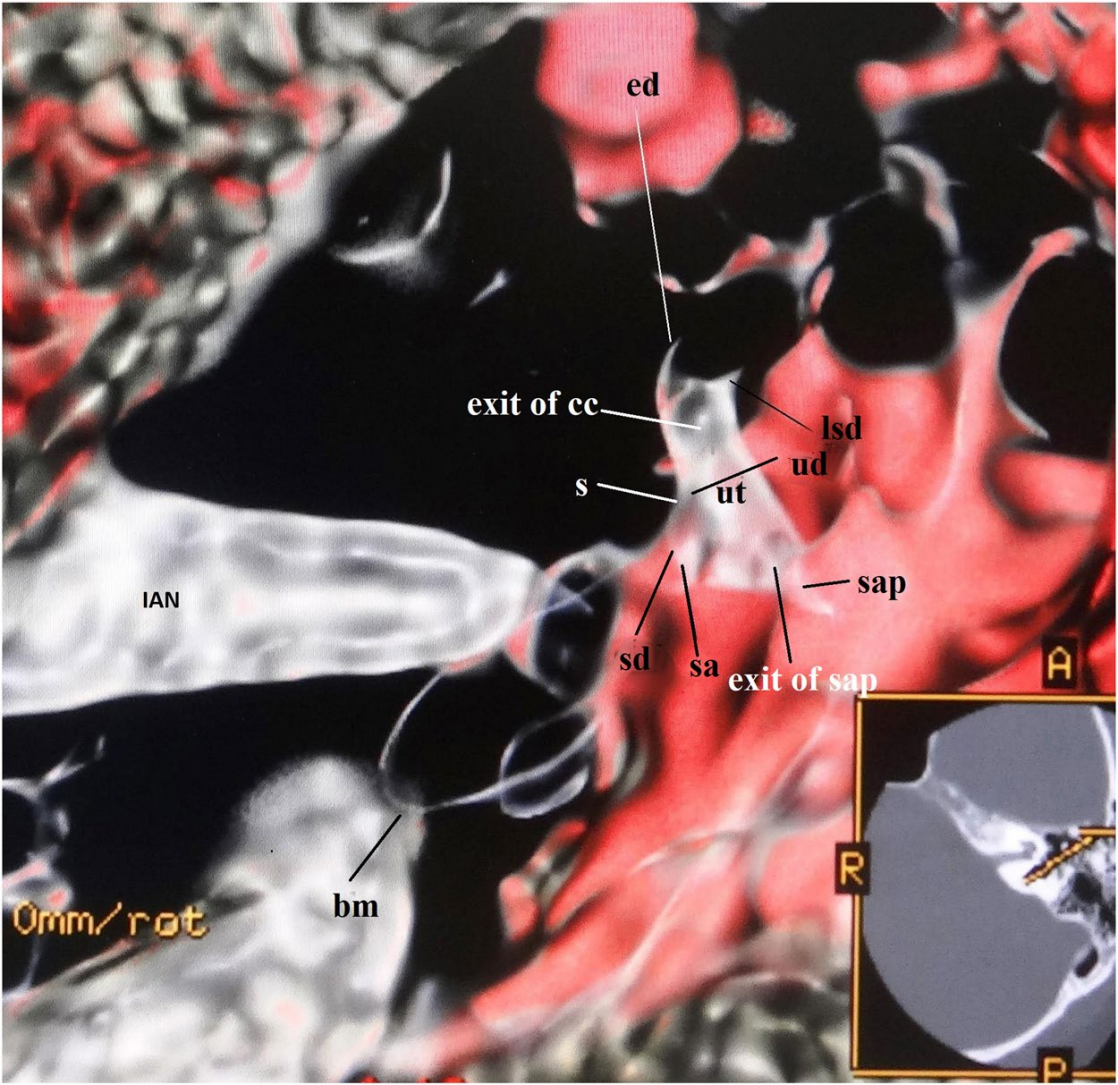
Superioanterior view of the utricle and the saccule in a 59-year-old male. This image is produced from a downward curve with a merged color algorithm under 40% opacity value. The major of the utricle lies parallel to the plane of the lateral semicircular ampulla. The endolymphatic duct and the sinus connect to the utricle and the saccule. Abbreviations: ut = utricle, sa = saccule, ed = endolymphatic duct, s = sinus, sd = saccular duct, utricular duct = ud, cc = common crus, lsd = lateral semicircular duct, lap = lateral ampulla, sap = superior ampulla, bm = basilar membrane, IAN = Internal Auditory Nerve.

The utricle, saccule, sinus, and endolymphatic duct as fibrotic microstructures could be observed. The utricular and saccular ducts were connected to the sinus. They made a triangle shape. The sinus led to the endolymphatic duct

### Quantitative image analysis

Table 1 showed the measurement results of this study. The histological measurements of the previous papers were shown in Table 2. The comparison analysis of the measurements obtained with our results and the previous ones was as follows.

**Table 1:** Dimensions of the utricle, saccule, and lateral semicircular duct, and the angle between the utricle and saccule.

Case no/Age/Sex		Utricle			Saccule			Lateral semicircular duct	
		Width (mm)	Length (mm)	Area (mm^2^)	Width (mm)	Length (mm)	Area (mm^2^)	Angle (degree)	Diameter (mm)
(1) 69/F	Rt	2.0	2.7	4.2	1.2	2.8	2.6	75.0	0.3
	Lt	2.0	2.7	4.2	1.2	2.7	2.5	75.2	0.3
(2) 26/F	Rt	2.2	2.6	4.3	1.0	2.9	2.3	73.0	0.3
	Lt	2.1	2.6	4.3	1.0	2.7	2.1	73.0	0.3
(3) 47/F	Rt	2.1	2.4	4.0	1.1	2.8	2.4	73.0	0.2
	Lt	2.0	2.4	3.8	1.0	2.9	2.3	74.2	0.2
(4) 62/F	Rt	2.1	2.4	4.0	0.9	3.0	2.1	75.0	0.3
	Lt	2.1	2.4	4.5	1.0	3.0	2.4	74.0	0.3
(5) 64/F	Rt	2.2	2.6	4.3	1.1	3.1	2.7	74.0	0.3
	Lt	2.1	2.6	4.2	1.1	3.1	2.7	74.5	0.3
(6) 61/M	Rt	2.0	2.7	4.2	1.0	2.7	2.1	78.0	0.3
	Lt	2.0	2.7	4.1	1.0	2.7	2.1	77.0	0.3
(7) 26/M	Rt	2.1	2.5	4.1	1.0	2.5	2.0	74.0	0.2
	Lt	2.1	2.5	4.1	1.0	2.6	2.0	75.0	0.2
(8) 37/M	Rt	2.0	2.6	4.4	1.1	2.7	2.3	74.0	0.3
	Lt	2.0	2.6	4.2	1.0	2.7	2.1	74.5	0.2
(9) 45/M	Rt	2.1	2.7	4.4	1.1	2.8	2.4	74.0	0.3
	Lt	2.0	2.7	4.4	1.1	2.9	2.5	74.0	0.3
(10) 59/M	Rt	2.0	2.8	5.6	1.1	3.0	2.6	74.0	0.3
	Lt	2.0	2.8	5.6	1.1	3.0	2.6	75.0	0.3
Mean 49.6		2.06	2.59	4.19	1.06	2.83	2.34	74.52	0.28
SD		0.07	0.18	0.17	0.76	0.17	0.24	1.23	0.04

**Table 2:** The histological dimensions of the utricle, saccule, and lateral semicircular duct, and the angle between the utricle and saccule.

Reporter	(year)	(age)	Utricle			Saccule			LSD*	
			Width	Length	Area	Width	Length	Area	Angle	Diameter
			(mm)	(mm)	(mm^2^)	(mm)	(mm)	(mm^2^)	(degree)	(mm)
Corversa *et al*.^20^	(1958)		2.1	2.8	4.2	1.2	2.2	2.2	72	
Beck & Rader^21^	(1963)		2.0	2.7	—	1.3	2.4	—	—	
Igarashi^22^	(1967)							2.08		
Rosenhal^23^	(1977)		2.2	2.8	4.29	1.2	2.6	2.24		
Watanuki & Schuknecht^24^	(1976)				3.57			2.20		
Igarashi *et al*.^25^	(1983)				3.271			2.188		
Takagi & Sando^26^	(1988)	(5 ma)	2.03	2.99	3.96	1.36	2.84	2.69	74.4	
		(14 yr)	2.38	3.47	5.18	1.64	2.90	2.95	68.2	
		(70 yr)	2.71	3.26	5.79	1.38	2.40	2.76	81.5	
		(76 yr)	2.40	2.87	4.82	1.32	2.26	2.19	83.8	
Curhoys & Oman^27^	(1987)									0.24–0.30
Mean			2.28	2.98	4.39	1.34	2.51	2.39	75.98	0.27
SD			0.24	0.28	0.84	0.15	0.27	0.32	6.53	0.03
Number (n)			7	7	8	7	7	9	5	12
**This study**										
Mean		49.6	2.06	2.59	4.19	1.06	2.83	2.34	74.5	0.28
SD		15.4	0.57	0.18	0.17	0.76	0.24	0.23	1.23	0.04
Number			20	20	20	20	20	20	20	20

*Lateral Semicircular Duct.

#### Utricle

It is no statistically significant association in the width and the area, except for the length between our results and those of previous histological reports. The results were as follows; the width was NS (t = −1.406, f = 23.792, p = 0.173). the area was NS (t = 2.635, f = 7.231, p = 0.545), the length was p < 0.01 (t = 3.444, f = 7.810, p = 0.009).

#### Saccule

There was no statistically significant association between them as follows; the width: NS (t = 1.563, f = 22.579, p = 0.132), the length: NS (t = −1.639, f = 9.057, p = 0.135) and the area: NS (t = 0.442, f = 11.885, p = 0.680).

#### The Angle between utricle and saccule

It is no statistically significant association as NS (t = 0.385, f = 4.071, p = 0.720).

#### Lateral semicircular duct

It is no statistically significant association as NS (t = −0.707, f = 29.991, p = 0.485).

### Analysis of the age-related changes in the vestibular membranous labyrinth

There was no correlation between the membranous CT value and the age group in gender differences as follows: both genders; r (r^2^): 0.172 (0.030), males; −0.194 (0.038), and females: 0.125 (0.016)

The statistical results of the age-related changes in each organ of the vestibular membranous labyrinth were shown in Table 3. The summarized results were as follows.

**Table 3:** Simple linear regression and correlation between each organ measured value and age.

	Both Genders	Females	Males
**Utricular area**			
Linear equation	y = 13.835 X − 8.369	y = −1.979 X + 63.240	y = 69.744X − 248.718
r (r^2^); F0 (fi:f2)	0.154 (0.024); 0.436: (1;:8)	−0.025 (0.001); 0.004 (1:8)	0.658 (0.434); 6.122 (1:8)
Significance (Precision P)	NS (0.517)	NS (0.946)	p ≺ 0.05 (0.038)
**Saccular area**			
Linear equation	y = 38.950 X − 41.544	y = 46.713 X − 58.979	y = 33.710X − 31.258
r (r^2^); F0 (f1:f2)	0.594 (0.352); 9.795 (1:18)	0.619 (0.384); 4.972 (1:8)	0.507 (0.251); 2.705 (1:8)
Significance (Precision P)	p < 0.01 (0.006)	NS (0.056)	NS (0.131)
**Angle between the saccule and the utricle**			
Linear equation	y = 38.284 X − 40.365	y = 16.237 X − 1149.401	y = 5.339X − 354.545
r (r^2^); F0 (f1:f2)	0.567 (0.322); 8.530 (1:18)	0.847 (0.718); 20.340 (1:8)	0.545 (0.297); 3.376 (1:8)
Significance (Precision P)	p < 0.01 (0.009)	p < 0.01 (0.002)	NS (0.103)
**Lateral Semicircular Duct**			
Linear equation	y = 80.000 X + 27.600	y = 82.500 X + 30.500	y = 227.619X − 15.857
r (r^2^); F0 (f1:f2)	0.231 (0.053); 1.011 (1:18)	0.211 (0.045); 0.373 (1:8)	0.788 (0.622); 13.141 (1:8)
Significance (Precision P)	NS (0.328)	NS (0.568)	p < 0.01 (0.007)

#### Utricular area

There was a statistical relationship in males, but not in females.

#### Saccular area

There was a statistical relationship between both genders. Females suggested a relationship as p = 0.056.

#### The angle between the utricle and the saccule

There was a statistical correlation in both genders.

#### The width of the lateral semicircular duct

A significant statistical correlation was in males, but a very weak correlation in females.

## Discussion

The created 3D vestibular membranous labyrinth image is almost consistent with the previous histological ones because of its shape and dimensions. There is a statistically significant difference in the length of the utricle between our measurements and the previous histological ones. However, we selected seven higher measurement results (a mean ± SD: 2.73 ± 0.05 mm; n = 7), and the statistical difference test was performed again. The Welch test showed NS (t = −2.325, f = 6.382, p = 0.057). Therefore, these generated 3D microstructure images can be considered to approximate the *in vivo* membranous labyrinth. The precise volume rendering technique is suitable to delineate the complex microanatomy of the membranous labyrinth. It depends on the optimal algorithms. Therefore, the different results can be obtained from them and the created microanatomical images appear qualitatively different. These algorithms provide a translucent view, thus giving a good impression of the spatial relationships of all microanatomical structures. For the optimal imaging result, it is necessary to use the different visualization techniques for each anatomical tissue in the membranous labyrinth.

In a threshold method, volume rendering causes tissues to fade from visible to invisible when thresholding completely hides or shows tissues of a certain density. However, partial volume effects can generate a phantom voxel value within the threshold range selected at the interface between dark and bright regions. In this study, the partial volume effect does not need to be considered, as the osseous vestibular values (a mean ± SD: 284.65 ± 47.80HU) were over the threshold value (under 160HU).

However, this technique has some problems with an algorithm, a threshold and, an opacity. There is a positive statistical correlation between the membranous CT density and the opacity values. So, the optimal opacity curve algorithm will fit the upward curve. However, to create all 3D membranous structures, we need to use the optimal opacity curve algorithm separately for each microanatomical structure. That is, creating different 3D microanatomical images requires the use of different combined algorithms separately. And more, to create the precise 3D microanatomical image, the optimal opacity should be adjusted to the optimal threshold for the target organ. These variable values also will lead to more precise microanatomical information. At this point, further study is needed.

The second problem is image resolution. In these minute microanatomical areas, image resolution is the most concerning issue. The resolution in this study is 0.18 × 0.18 × 0.5 mm^3^. This value is relatively rough for describing the microstructures of the membranous labyrinth. Therefore, a smaller FOV and thinner slice thickness should be used. However, this problem is that it depends on the capabilities of the CT machine.

The third problem is the depiction of the otoliths from this technique. It is impossible to create the otoliths using these created algorithms. Because, the otoliths are made of calcium carbonate (CaCO3), and the CT value of CaCO_3_ exceeds 140 HU (a mean ± SD: 189 ± 38.4)^29^. This value is higher than the membranous vestibular CT value (a mean ± SD: 110.446 ± 21.176 HU). So, we cannot create the otoliths from the algorithms under the defined threshold in this study. When investigating the otoliths, we need to create other algorithms with the optimal threshold value. The part of this study was published before as an abstract form^30^.

However, this technique is relatively simple and safe, and can be used for daily practice to know the membranous labyrinth. Because this technique using a conventional CT machine is low radiation dose exposure. The DLP was 40 to 55 mGy/cm. That is, the radiation dose was 0.06 to 0.08 mSv^31^. This radiation dose is about the same as a skull X-P^32^.

On the other hand, endolymphatic hydrops imaging from two MRI methods has greatly impacted the field of otology and has prompted an ongoing shift in the diagnostic paradigm for Meniere’s disease (MD)^3–5^. These MRI methods using GBCAs with a head coil can obtain only a bidimensional image of endolymphatic hydrops. Therefore, these methods have three disadvantages.

The first concern is the potential for toxicity of GBCAs. The mechanisms of GBCAs toxicity is gradually revealing from recent studies^6–8^. Rogosnitzky & Branch^6^ review gadolinium accumulation in various tissues of patients without renal impairment, including bone, brain, and kidneys. One of the most important age-related pharmacokinetic changes is decreased renal excretion of the drug. Older people generally have less muscle volume and less physical activity than younger adults, and therefore less creatinine production. So, their serum creatinine levels usually remain within the normal range despite a reduced glomerular filtration rate (GFR) in the elderly. Therefore, the contrast agent administration will be considered carefully, even if normal serum creatinine levels are maintained. Rah *et al*.^7^ state that intratympanic contrast injection can cause local toxicity in animal models. However, it may not be likely that a GBCA will lead to hair cell toxicity, as the estimated concentration in the inner ear after clinically tried intratympanic injection is far more diluted. Type I and type II hair cells decrease with age and are fitted by a linear regression model^33^. Hence, this method in the elderly may decrease hear cells. The newly added alert from the FDA in 2018 states that gadolinium can stay in the body for months to years after receiving these drugs during an MRI scan^8^. It is important to note that metabolism declines with age. If GBCAs administration is the best option for a given patient, a pretreatment approach that protects against toxicological endpoints may be a viable option.

The second concern is the low-resolution images of the endolymph or perilymph within the minute structures in the inner ear^3,4^. The image voxel volume for these methods is approximately 0.5 mm^3^. It is not suitable to visualize micro-changes in the vestibular membranous labyrinth. We think that this factor makes the inability to distinguish the two adjacent structures between the utricle and the saccule because of the same signal strength and no visible boundaries between them. Therefore, it is probable that the vestibular end-organs cannot be measured accurately than our method.

The third concern is whether these methods can be used as a new test for the symptom-based diagnostic criteria for MD^34^. Lopez-Escamez & Attyé^5^ review magnetic resonance imaging for the diagnosis of MD. Their significant concept from the imaging of endolymphatic hydrops is the realization that clinical MD likely only represents a partial match of patients with endolymphatic hydrops and clinical symptoms. Because the patients for the typical clinical definition of MD often do not fit the endolymphatic hydrops imaging. They state that endolymphatic hydrops can be reliably measured in the saccule using the saccule to the utricle area ratio (SURI). This implies that the saccule plays a major role in MD. The saccule may not expand in some of MD cases. The reason for this discrepancy is that the ability to measure a microanatomical structure does not depend on the patient’s symptoms but technical factors. Therefore, these MR methods cannot use as a new diagnostic tool for MD. To solve this problem, it is necessary to create high-resolution MR imaging techniques. However, their review sheds light on the complexities of MRI in the diagnosis of MD but it is clear more research is needed, including the use of our method.

Our measurement results imply the age-related changes in the vestibular membranous labyrinth. However, there are no age-related changes in the membranous vestibular CT value in both genders. In other words, the CT value of the vestibular substances is almost constant regardless of age. On the other hand, the dimensions of the utricle, the saccule, and the lateral semicircular duct show the age-related changes. And these changes suggest that they may correlate with some of the changes in physiological aging. The angle between the utricle and the saccule increases with age. This means that the vestibular membranous labyrinth will deform in each organ with age, and then the angle between them will increase. In this study, the saccule expands with age in females, and the utricle expands with age in males. These results imply that these minute structural changes may be correlated with the physiological findings from the several previous articles of ocular and cervical vestibular evoked myogenic potentials (VEMPs), and of gait studies^12–18^. The origin of cVEMP is considered to be the saccular function, and the origin of oVEMP is considered to be the utricular function. The elderly ocular and cervical VEMPs amplitude are small. cVEMP response latencies are also longer and depend on a greater extent on stimulus volume to generate an effective response in the elderly. oVEMP response rate has been shown to decrease with normal aging. The elderly show less ocular counter roll during slow roll tilt and also in response to electrical vestibular stimulation consistent with decreased utricular responsiveness. Although all studies find that the latency increases with age, females may be less likely to show this latency change than males^12,13^. These physiological findings may suggest a correlation with our measurement results that the utricle expands with age in males, not in females. Regarding walking, the decrease in walking speed with age is slower in males than in females^14^. Makiura *et al*.^15^ reported that stride-to-stride time variability (STV) was significantly increased in females compared to males, and females exhibited significantly higher root-mean-square (RMS) of vertical and anterior-posterior axes and lower auto-correlation coefficient (AC) of vertical axes than males. These results suggest that females have lower gait stability than males. The reactive control mechanism, distinct from the proactive control balance mechanism, mainly depends on somatosensory and vestibular systems to determine the extent and the stimulation type and to trigger a properly scaled postural response^16^. Females have lower AC of vertical axes than males. This phenomenon is interpreted as being associated with a relatively saccular insufficiency in females. Although age-related vestibular losses are noted for both genders, oVEMPs are not associated with gait speed in both genders^17^. These results suggest that gender differences in walking speed with age may be caused by the saccular function. These suggest a correlation with our results that the saccular dimension with age increases in females but not in males. For the lateral semicircular duct, investigations on the effects of normal aging on caloric response find a significant increase in response for middle-aged groups followed by a slow decline with increasing age^18^. In this study, the width of the lateral semicircular duct in the vicinity of the ampulla is significantly related to male aging and very weakly related to female aging. Thus, it will imply that the endolymphatic flow velocity in the semicircular duct slows with age due to its dilation.

These created *in vivo* 3D vestibular membranous labyrinth images have the potential to reveal the interrelationship of each organ in the *in vivo* vestibular microanatomy. The *in vivo* 3D microanatomical image changes may resemble the results of previous physiological works. Therefore, these created images may be useful for the study of physiological phenomena in the vestibular membranous labyrinth.

In conclusion, the 3D microanatomical imaging technique can be used for the precise microanatomical study of *in vivo* 3D vestibular membranous labyrinth. The micromorphological changes demonstrated by this method may represent the age- and gender-related changes in balance. The created 3D microanatomical image suggests physiological phenomena. We believe that this 3D technique will improve the understanding when dealing with various etiopathology and treatments of the vestibular end-organs.

## Participants and Methods

### Participants

This study was conducted under the approval of Tanioka Clinic review board with the 1964 Helsinki Declaration and its later amendments or comparable ethical standards. All participants gave written informed consent.

Imaging data from 20 normal ears of 10 participants (10 ears of 5 females, mean age: 53.6 years; 10 ears of 5 males, mean age: 45.6 years) who visited for their regular check-ups and wished to inspect the head and temporal bone regions were obtained. These participants had no known affliction of the temporal bone and no clinical history of hearing and balance problems, and normal findings on both CT exams and physical exam by their check-ups. For histological subjects, the histological results of previous works of literature were used^19–28^.

### CT protocol

All examinations were performed with a spiral CT scanner (ProSpeed AI; General Electric Systems, Milwaukee, Wis, USA). The section thickness was 0.5 mm. A voltage of 120 kV and a current of 60–120 mA were used. The axial images of the temporal bone were reconstructed with a high-resolution bone algorithm in steps of 0.5 mm and a FOV of 96 × 96 mm^2^ using a 512 × 512 matrix.

### Volume rendering and opacity value

Volume rendering can be used to show both objects at the same time. We seek to display a membranous tissue among the surrounding osseous vestibule. By assigning a low opacity to the osseous vestibule and a high opacity to the membranous tissue, the membranous tissue becomes clearly visible among its semi-transparent surroundings. Where thresholding either entirely shows or hides tissue of a given density, volume rendering shows the tissue to fade from visible to invisible. Assigning the same density to both makes them indistinguishable. When the density of the outer object gradually decreases, the inner object becomes visible. That is, the density ratio between them is the highest opacity.

In this study, the opacity value was calculated by the formula.

$${\rm{Opacity}}\,{\rm{value}}( \% )={\rm{membranous}}\,{\rm{density}}({\rm{HU}})/{\rm{osseous}}\,{\rm{density}}({\rm{HU}})\times 100$$

### Opacity curve algorithm

Tissue-specific images with a model defined in terms of opacity were created by various opacity curve algorithms. We created optimal combination algorithms suitable for imaging each membranous organ as follows^9–11^.

A. An upward opacity curve using a merged color algorithm
  An upward curve algorithm shows bright voxels. It may be used to display bright structures, such as bone or vessels in CT data sets. Therefore, this is suitable for the soft tissues on the osseous lamina.
B. An upward opacity curve combining a merged color and a step algorithm
  A step curve algorithm displays a surface shading image. This is a step curve with only one control point. It allows us to obtain a surface shading rendering on the volume rendering views. This algorithm fits imaging semicircular ducts.
C. A trapezoid opacity curve using a non-merged colored algorithm
  A trapezoid curve algorithm shows voxels within a threshold range. This curve type is used to display structures with voxel values within a range. This algorithm is common for creating the membranous labyrinth.
D. A downward opacity curve using a merged colored algorithm

A downward curve algorithm shows dark voxels. This curve type can be used to display dark structures, such as the organs of Corti within the cochlear duct, the saccular, utricular, and ampullary organs.

### Setting volume rendering parameters

Using a transparent volume-based technology, we defined parameters to carry on the algorithms from a threshold method. If the brightness value was set constant under the defined threshold value, the created image depended on the opacity value. In this study, the brightness value was defined as the constant value of 100%. Therefore, to create a 3D image, we must determine the optimal threshold and opacity values from vestibular CT values.

### Measurements of vestibular CT Value

To define the optimal threshold value, the radiodensity of both the whole vestibule along with the bone wedge (osseous vestibule) and the soft tissue component (membranous vestibule) within the osseous vestibule were measured in Hounsfield units (HU) using 0.5-mm-thick axial high-resolution temporal CT image slices at the level through the oval window, the cochlea, and the vestibule. The imaging conditions were 2000 HU in window width, and 200 HU in window level. The membranous vestibule was composed of the soft tissues, the nerve fibers with the myelin containing the lipid component, the fluids, and the fat. Therefore, the lowest CT value was fat^35^ of the lipid component. So, the lowest threshold and opacity values were −100 HU and 0%, respectively. Table 4 showed the measurement results and opacity of two parts of the vestibule.

**Table 4:** Osseous and Membranous CT values and Opacity.

Case	Age	Sex	Side	Osseous Vestibule	Membranous Vestibule	Ratio (Opacity)
				(HU)	(HU)	Membranous/Osseous (%)
1	69	F	Rt	326.68	84.38	25.83
			Lt	245.72	119.82	48.88
2	26	F	Rt	262.27	92.12	35.12
			Lt	250.40	139.36	55.65
3	47	F	Rt	217.99	89.79	41.19
			Lt	228.83	78.31	34.22
4.	62	F	Rt	209.37	104.52	49.92
			Lt	247.33	120.13	48.57
5.	64	F	Rt	277.73	119.60	43.06
			Lt	306.60	126.51	41.26
6.	61	M	Rt	381.47	92.31	24.20
			Lt	384.17	112.20	29.21
7.	26	M	Rt	275.07	86.92	31.60
			Lt	304.59	115.62	37.96
8.	37	M	Rt	281.06	18.03	41.99
			Lt	285.38	99.63	34.91
9.	45	M	Rt	273.39	158.65	58.03
			Lt	303.56	111.74	36.81
10.	59	M	Rt	339.25	126.66	37.34
			Lt	292.08	109.85	37.61
Mean	49.6			284.65	110.31	39.67
SD	15.8			47.80	19.93	9.13

### Threshold and opacity values

Since the statistical phenomena in physical measurements followed the normal distribution, our measurement results must be normally distributed. A goodness-of-fit test was used to examine the normal distribution of both the membranous vestibular CT values and the opacity ones. The results showed the normal distribution. Both normal probability graphs showed nearly linear. The results of these categories of rank, mean, variance, SD, degree of freedom, χ² value, significance, and precise p-value were suitable for normal distribution. The scatter diagram and the linear regression between the membranous vestibular and the opacity values were obtained from Table 4. The linear equation was Y = 0.181X + 20.430 (Y = the percentage opacity value, X = the membranous vestibular CT value, r: 0.771, p < 0.001). Although the opacity should be defined from the membranous CT value, in this study, we used the mean opacity value of 40% for simplicity. The highest threshold value was 160 HU from Table 4.

### Postprocessing

After transferring the image data to a CT workstation (GE Healthcare; Chicago, IL, USA), 3D visualization based on the interactive direct volume rendering was made using GE Advantage Navigator software Ver. 2. Direct volume rendering considers some of the image data, so roughly simple explicit segmentation using a cutting-plane method^36^ before the visualization process was required. As a result of this software, both color and opacity values were adjusted interactively to delineate all structures related to the membranous vestibule in real-time. After an appropriate setting using the front cut by a cutting-plane method for optimal delineation of the target structures was defined on axial, coronal, and sagittal projection, the color and opacity table can be stored and used for further studies. The user ensured the intuitive manipulation of any object in real-time. The software allowed both distance measurements and areas directly within the 3D scene. This software was almost the same as the open-source OsiriX software (available at http://www.osirix-viewer.com).

### Image analysis

Two specialists evaluated the created images by both qualitative and quantitative analyses. One radiologist specialized in head and neck imaging had 30 years of experience, and one otologist had 40 years of experience.

### Qualitative image evaluation

Qualitative image assessment was assessed by visualization as the following structures. 1: the utricle (ut), 2: the saccule (sa), 3: the superior ampulla (sap). 4: the lateral ampulla (lap). 5: the posterior ampulla (pap), 6: the superior semicircular duct (ssd), 7: the lateral semicircular duct (lsd), 8: the posterior semicircular duct (psd), 9: the common crus (cc), 10: the sinus (s), 11: the saccular duct (sd), 12: the utricular duct (ud), 13: the endolymphatic duct (ed). 14: the facial nerve (fn). 15: the vestibular nerve (vn). 16: the cochlear nerve (cn). The inside of parentheses is an abbreviation.

### Quantitative image analysis

#### Measured dimensions

Utricle. The width of the utricle was measured from the anterior border to the posterior edge, and the length was between the edges of the anterior duct and the common crus in the superior view like Fig. 2.

Saccule. The longitudinal axis was taken to be a line along the surface that seemed to represent the median of the saccule in the frontal view as in Figs. 1 and 3. The width of the saccule was determined by the widest perpendicular line to the longitudinal line.

Lateral semicircular duct. The width of the lateral semicircular duct was measured in the vicinity of the ampulla like Fig. 2.

The angle between utricle and saccule. The angle between them was measured in a 3D lateral view.

### Analysis of the age-related changes

We examined the relationships between each measurement value and the age group as follows. 1. the membranous CT value. 2. the areas of the utricle and the saccule. 3. The angle between the utricle and the saccule. 4. the width of the lateral semicircular duct near the ampulla.

### Statistical analysis of measurements

The statistical correlations between the CT value of the membranous vestibule, each organ in the membranous vestibule, and the age group in genders were calculated by univariate linear regression analysis and expressed by Pearson’s correlation coefficient. To determine whether the measured results between this study and the previous histological data were equal, we carried on the Welch test. A p-value of < 0.05 was regarded as significant. Data were analyzed statistical software available through Microsoft Excel 2013 (Microsoft Corporation Redmond, Washington).
